# Visual tracking of viral infection dynamics reveals the synergistic interactions between cucumber mosaic virus and broad bean wilt virus 2

**DOI:** 10.1038/s41598-023-34553-6

**Published:** 2023-05-04

**Authors:** Min-Jun Kwon, Sun-Jung Kwon, Myung-Hwi Kim, Boram Choi, Hee-Seong Byun, Hae-Ryun Kwak, Jang-Kyun Seo

**Affiliations:** 1 Department of International Agricultural Technology, Seoul National University, Pyeongchang, 25354 Republic of Korea; 2 Institutes of Green Bio Science and Technology, Seoul National University, Pyeongchang, 25354 Republic of Korea; 3 Department of Agricultural Biotechnology, Seoul National University, Seoul, 08826 Republic of Korea; 4 Crop Protection Division, National Institute of Agricultural Sciences, Rural Development Administration, Wanju, 55365 Republic of Korea

**Keywords:** Pathogens, Virology

## Abstract

Cucumber mosaic virus (CMV) is one of the most prevalent plant viruses in the world, and causes severe damage to various crops. CMV has been studied as a model RNA virus to better understand viral replication, gene functions, evolution, virion structure, and pathogenicity. However, CMV infection and movement dynamics remain unexplored due to the lack of a stable recombinant virus tagged with a reporter gene. In this study, we generated a CMV infectious cDNA construct tagged with a variant of the flavin-binding LOV photoreceptor (iLOV). The iLOV gene was stably maintained in the CMV genome after more than four weeks of three serial passages between plants. Using the iLOV-tagged recombinant CMV, we visualized CMV infection and movement dynamics in living plants in a time course manner. We also examined whether CMV infection dynamics is influenced by co-infection with broad bean wilt virus 2 (BBWV2). Our results revealed that no spatial interference occurred between CMV and BBWV2. Specifically, BBWV2 facilitated the cell-to-cell movement of CMV in the upper young leaves. In addition, the BBWV2 accumulation level increased after co-infection with CMV.

## Introduction

Cucumber mosaic virus (CMV; genus *Cucumovirus*, family *Bromoviridae*) is one of the most agriculturally significant viruses owing to extensive economic and yield losses^1^. CMV has successfully dispersed worldwide, infecting more than 1200 species, and is most prevalent in various horticultural crops, including pepper, tomato, cucumber, and lettuce^2^. Generally, CMV induces severe symptoms in its host plants, such as mosaic, leaf size reduction, and stunted growth, and causes an average yield loss of 10–20% in various crop plants^2^. In particular, CMV is endemic in most pepper growing areas globally^3^. Yield losses due to CMV infection in pepper ranged from 10.84 to 50.51% during the period 2010–2012 in Bangladesh^4^. In over 100 years since its discovery^5,6^, CMV has been extensively studied as a model RNA virus to understand various aspects of the virus, including its pathogenicity, evolution, replication, virion structure, and gene functions^2,7,8^. However, the dynamics of the systemic movement of CMV in infected plants remains unexplored.

The CMV genome comprises three positive-sense single-stranded RNAs^2,8^. RNA1 and 2 encode the viral replicase proteins, 1a and 2a, respectively. RNA2 also contains a second open reading frame (ORF) encoding 2b, which is an RNA silencing suppressor. In particular, 2b is expressed from RNA4A, a subgenomic RNA derived from RNA2 during virus replication^9^. A 2b gene deletion mutant of CMV (CMVΔ2b) that systemically infects *Nicotiana benthamiana*, *N. tabacum*, and *Arabidopsis thaliana* ecotype Col-0 induces significantly attenuated symptoms in these host plants, suggesting that 2b is involved in the development of symptoms^10,11^. RNA3 encodes two proteins, the 3a (movement protein; MP) and the coat protein (CP); CP is expressed from RNA4, a subgenomic RNA derived from RNA3^12^. Infectious cDNA clones of the genomic RNAs of various CMV strains have been constructed under the control of either a T7 promoter for in vitro transcription or the cauliflower mosaic virus 35S promoter and widely used to study the molecular biology of CMV^8,11,13–16^. The CMV infectious cDNA clones have also been engineered as viral vectors for foreign gene expression or gene silencing in plants^17–23^.

A range of techniques have been employed to examine the dynamics of cell-to-cell and systemic movement of plant viruses. However, the majority of them use destructive approaches, such as in situ hybridization and immunogold labeling, for the detection of viral nucleic acids and proteins, respectively, in plant cells and tissues^24–27^. Recombinant viruses tagged with fluorescent proteins and non-fluorescent reporters have been generated for various plant viruses for which their infectious DNA clones have been developed^28–33^. These approaches using tagged viruses have enabled the observation of the time-course dynamics of viral infection and movement in living plants. However, the stability of the inserted tag in the genome of progeny viruses should be addressed for accurate tracking of viral infection and movement because inserted foreign sequences can often be partially or entirely removed during the rapid and error-prone replication of the viral genome^34–36^.

Although green fluorescent protein (GFP)-based fluorescent proteins have been used extensively as reporters of viral infection and movement^28,30,35^, tagging with GFP is unstable due to the small genome capacity of some viruses, including CMV^18,19,21,37^. To fluorescently tag CMV, an infectious cDNA clone of RNA2 was modified to incorporate an ORF encoding a GFP gene in a variety of configurations^21^. Among the recombinant RNA2 constructs incorporated with GFP, one construct containing an intact 2a ORF and an ORF encoding the N-terminal two-thirds of 2b fused in-frame with GFP was able to systemically infect *Nicotiana benthamiana* plants and express GFP on inoculated and systemic leaves until 14 days post-inoculation (dpi). However, after 14–21 dpi, no GFP fluorescence was detected on newly emerging leaves. RT-PCR analysis revealed a loss in the integrity of the GFP tag in the genomes of progeny viruses^21^. As an alternative to GFP, a flavin-based fluorescent protein variant derived from the light, oxygen, or voltage-sensing domain of phototropin (iLOV) was developed and used as a reporter for plant virus infections^32^. As the size of the iLOV gene (375 bp) is considerably smaller than that of GFP (~ 720 bp), iLOV is predicted to have reduced constraints on CMV genetic capacity compared with GFP. Correspondingly, a recent study demonstrated that tagging of CMV with iLOV was stable until 28 dpi in the upper leaves of inoculated *N. benthamiana*, allowing for the long-term monitoring of viral distribution in living plants^37^.

Mixed infections with two or more viruses are common in crop fields^3,38^. Broad bean wilt virus 2 (BBWV2; genus *Fabavirus*, family *Secoviridae*) that commonly co-infects crop plants with CMV is another prevalent virus in pepper fields in South Korea^3,38^. Both CMV and BBWV2 are transmitted by aphids in a non-persistent manner^39,40^. Previous field surveys showed that more than 50% of the collected pepper samples were mixed-infected with CMV and BBWV2, and this double infection caused more severe damage in pepper plants than single infection with each virus through synergistic symptom expression^3,38^. Nevertheless, the molecular mechanisms underlying the synergistic interactions between CMV and BBWV2 in infection dynamics remain poorly understood.

In this study, we aimed to examine the time-course infection dynamics of CMV in living plants. We first generated a recombinant CMV tagged with iLOV (CMV-iLOV) using the similar strategy as described previously^37^ and confirmed the stability of the iLOV insertion into the CMV genome for long-term observations. Using CMV-iLOV, we examined the time-course infection dynamics of CMV in *N. benthamiana* plants at the whole plant level. We also investigated whether CMV infection dynamics was influenced by co-infection with BBWV2. Based on these approaches, we successfully visualized the time-course infection dynamics of CMV in single or mixed infection with BBWV2.

## Materials and methods

### Plant growth, viral sources, and inoculation

*Nicotiana benthamiana* plants were grown in an insect-free growth chamber with a cycle of 16 h of light at 26 °C and 8 h of darkness at 24 °C. Full-length infectious cDNA clones of the CMV GTN strain (CMV-GTN) and BBWV2 RP1 strain (BBWV2-RP1) were used as viral sources^15,30^ and engineered to generate fluorescently tagged viral constructs. Infectious cDNA clones of CMV and BBWV2 and their derivatives were inoculated by *Agrobacterium*-mediated infiltration (agroinfiltration) into leaves of two-week-old *N. benthamiana* plants, as previously described^14,15,41^. For serial passage experiments, crude sap was obtained from L4 leaves [the leaf positions were designated sequentially from inoculated leaves (L0) to upper leaves] of *N. benthamiana* infected with CMV-iLOV at 9 dpi in each passage and used for mechanical inoculation^42^. We have permission to collect the plant samples used in the study and all methods comply with local and national guidelines.

### Construction of fluorescently tagged CMV and BBWV2 constructs

**Figure 1:**
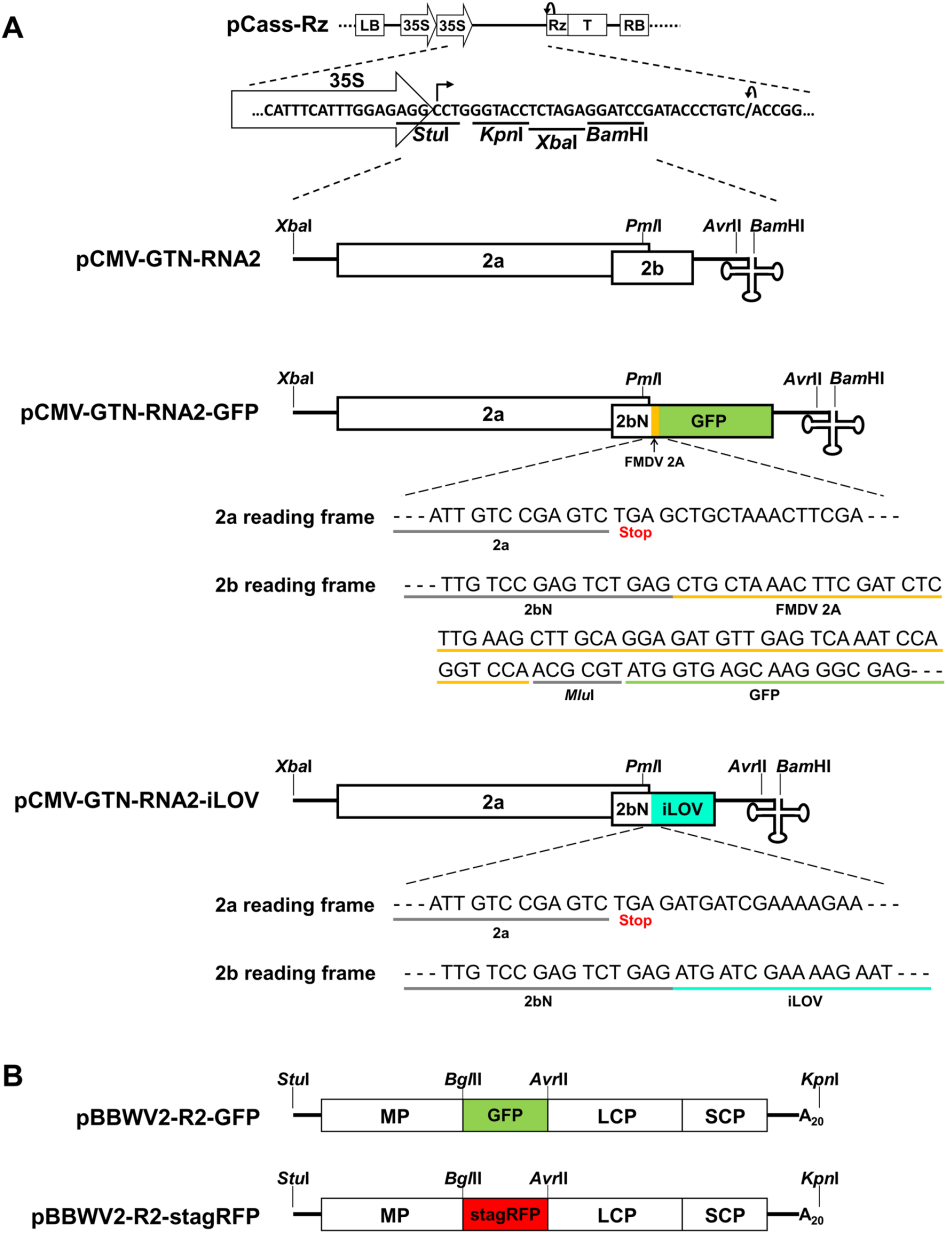
Schematic representation of viral infectious cDNA constructs used in this study. (**A**) Schematic representation of an infectious cDNA clone of CMV-GTN RNA2 pCMV-GTN-RNA2^15^ and fluorescently tagged derivatives. The pCass-Rz vector contains, in sequential order, a T-DNA left border (LB), a double 35S promoter, multiple cloning sites (*Stu*I, *Kpn*I, *Xba*I, and *Bam*HI), a cis-cleaving ribozyme sequence (Rz), a 35S terminator (T), and a T-DNA right border (RB). Open reading frames (ORFs) are indicated by rectangles. Restriction enzyme cleavage sites used to engineer the constructs are shown. pCMV-GTN-RNA2-GFP contains an intact 2a ORF and an ORF encoding the N-terminal two-thirds of 2b (2bN) fused in-frame with the FMDV 2A sequence and GFP. pCMV-GTN-RNA2-iLOV contains an intact 2a ORF and an ORF encoding the 2bN fused in-frame with iLOV. (**B**) Schematic representation of fluorescently tagged BBWV2 RNA2 constructs. The GFP and stagRFP genes were cloned into pBBWV2-R2-OE^30^ using the *Bgl*II and *Avr*II sites. The resulting constructs were designated as pBBWV2-R2-GFP and pBBWV2-R2-stagRFP, respectively.

An infectious cDNA clone of CMV-GTN RNA2 (pCMV-GTN-RNA2; Fig. 1A) was engineered to construct CMV RNA2 derivatives tagged with either GFP or iLOV. A 1106-bp DNA fragment comprising the C-terminus of 2a (from the *Pml*I site), the 2A self-cleaving sequence of foot and mouse disease virus (FMDV), an *MluI* site, the GFP coding sequence, and the viral 3’ untranslated region (UTR) (to the *Avr*II site) (Fig. 1A and Supplementary Fig. S1) was synthesized (Macrogen, Korea) and inserted into pCMV-GTN-RNA2, which was digested with *Pml*I and *Avr*II. The resulting construct was named pCMV-GTN-RNA2-GFP (Fig. 1A). The combination of pCMV-GTN-RNA1 + pCMV-GTN-RNA2-GFP + pCMV-GTN-RNA2 was designated as CMV-GFP.

Similarly, a 608-bp DNA fragment comprising the C-terminus of 2a (from the *Pml*I site), the iLOV coding sequence, and the viral 3′ UTR (to the *Avr*II site) (Fig. 1A and Supplementary Fig. S2) was synthesized (Macrogen, Korea) and inserted into pCMV-GTN-RNA2, which was digested with *Pml*I and *Avr*II. The resulting construct was named pCMV-GTN-RNA2-iLOV (Fig. 1A). The combination of pCMV-GTN-RNA1 + pCMV-GTN-RNA2-iLOV + pCMV-GTN-RNA2 was designated as CMV-iLOV.

The pBBWV2-OE vector^30^ was engineered to generate a BBWV2 construct tagged with super-TagRFP (stagRFP)^43^. The stagRFP sequence was synthesized (Macrogen, Korea) and inserted into pBBWV2-R2-OE, which was digested with *Bgl*II and *Avr*II^30^. The resulting construct was named pBBWV2-R2-stagRFP (Fig. 1B) and used to examine the systemic spread of BBWV2 in *N. benthamiana* plants co-infected with CMV-iLOV. The combination of pBBWV2-RP1-R1 + pBBWV2-R2-GFP, previously designated as BBWV2-GFP^30^, was used for time-course observations of BBWV2 infection dynamics in single infections.

### Fluorescence imaging

Time-course observations of fluorescence signals of GFP and iLOV in living plants were performed using a FOBI fluorescence imaging system (NeoScience, Korea) equipped with a blue light source (excitation at 470 nm) and an emission filter (530 nm short-pass), which removes auto-fluorescence signals from chlorophyll. Whole-leaf fluorescence signals were observed using an IVIS Lumina III fluorescence imaging system (Perkin Elmer, USA) equipped with specific excitation/emission sets for iLOV (480/520 nm) and stagRFP (560/620 nm). Cellular fluorescence signals emitted by iLOV and stagRFP in plant leaves were observed using a Leica SP8 laser-scanning confocal microscope (Leica, Wetzlar, Germany) equipped with specific laser/filter combinations for iLOV (excitation at 476 nm, detection between 510 and 550 nm) and stagRFP (excitation at 568 nm, detection between 580 and 620 nm).

### RNA extraction, virion purification, and virus detection and quantification

RNA was extracted using the PureLink RNA Mini kit (Ambion, USA), according to the manufacturer’s instructions. To detect CMV infection and evaluate the stability of the reporter gene insertion in virus progeny, total RNA was extracted from the upper uninoculated leaves of *N. benthamiana* plants inoculated with CMV-GFP or -iLOV and analyzed by RT-PCR using a primer pair spanning the gene insertion region (5′-TGACAAACGTCGAACTCCAACT-3′ and 5′-AGCAATACTGCCAACTCAGCT-3′). To analyze the integrity of the iLOV gene in viral RNAs packaged into virions, CMV virions were purified from systemic leaves infected with CMV or CMV-iLOV based on a micropurification method^44^ and subjected to RNA extraction followed by reverse transcription (RT)-PCR using the above primer pair spanning the gene insertion region. To quantify the accumulation levels of CMV and BBWV2, RT followed by quantitative (RT-qPCR) was performed using the 2X SYBR Green Real-Time PCR Smart mix (Solgent, Korea) and iCycler iQ5 detection system (Bio-Rad, USA) with the following specific primers: CMV-R3-1752-Fw (5′-GTTGTATGATCTTTCGGCGA-3′) and CMV-R3-2057-Rv (5′-TTTCTCCACGACTGACCATT-3′) for CMV RNA3 detection, BBWV2-R1-RT-Fw (5′-TCACAGGTTATGCCGCTTGT-3′) and BBWV2-R1-RT-Rv (5′-TCACTCGTCCCAAGCTGTTC-3′) for BBWV2 RNA1 detection, and Nb-actin-qRT-Fw (5′-CGAGGAGCATCCAGTCCTCT-3′) and Nb-actin-qRT-Rv (5′-GTGGCTGACACCATCACCAG-3′) for actin mRNA detection in *N. benthamiana*^14,45^. The actin gene was used as an internal reference control for normalization of RT-qPCR results. Three biological and three technical replicates were analyzed per sample.

## Results

### Infectivity and integrity of recombinant CMV tagged with two different fluorescent reporters

**Figure 2:**
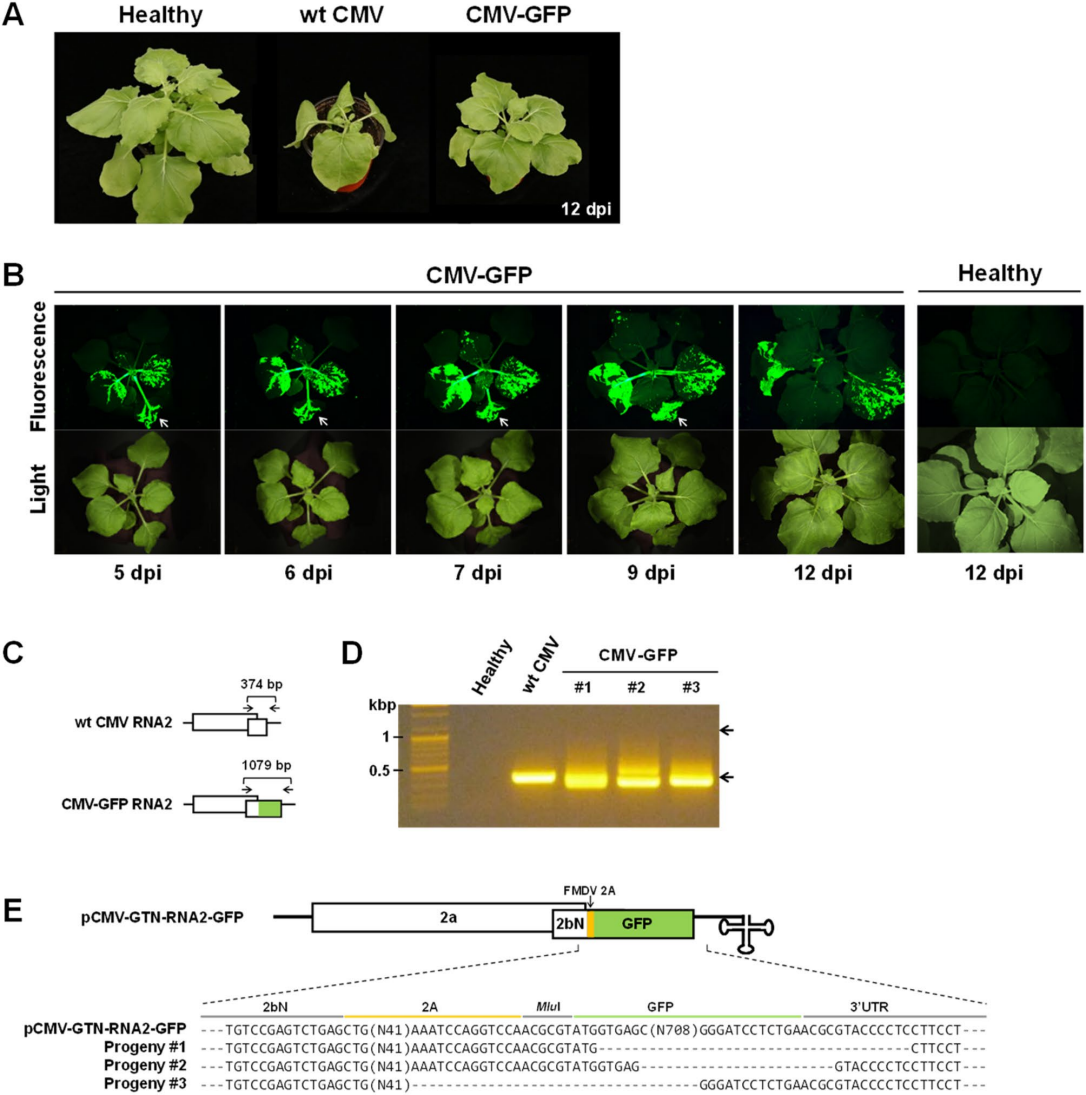
Infectivity, visual tracking, and stability of GFP-tagged CMV (CMV-GFP). (**A**) Virulence of infectious cDNA clones of wild-type (wt) CMV and CMV-GFP in *Nicotiana benthamiana*. At 12 days post-inoculation (dpi), wt CMV induced symptoms of severe stunting and leaf malformation, whereas CMV-GFP caused mild mosaic symptoms. (**B**) Time-course observation of CMV-GFP infection in *N. benthamiana*. *N. benthamiana* plants inoculated with CMV-GFP were observed using a FOBI fluorescence imaging system at 5, 6, 7, 9, and 12 dpi. Data shown are representatives of at least three independent experiments. (**C**) Schematic maps of wt CMV RNA2 and CMV-GFP RNA2. Arrows indicate the primer positions used for RT-PCR analysis, with the predicted PCR product sizes shown at the upper part. (**D**) RT-PCR analysis of the integrity of CMV-GFP. Total RNA was extracted from the upper youngest leaves of *N. benthamiana* plants inoculated with wt CMV or CMV-GFP at 12 dpi and analyzed by RT-PCR using the primers indicated in (**C**). No products with the predicted size for intact CMV-GFP (1079 bp) were amplified in RNA samples derived from the upper young leaves of *N. benthamiana* plants inoculated with CMV-GFP, whereas we detected products with a size similar to that of wt CMV (374 bp). Numbered lanes indicate three individual plants tested for CMV-GFP. Arrows point at RT-PCR products of expected sizes, as shown in C. The gel image has been cropped for clarity, and the original gel is presented in Supplementary Fig. S3. (**E**) Deletion of the GFP-coding sequence in CMV-GFP progeny viruses recovered from upper youngest leaves of *N. benthamiana* plants. Sequences of RT-PCR products of CMV-GFP progenies obtained in D were analyzed and compared with the parental sequence of pCMV-GTN-RNA2-GFP. Nucleotides adjacent to deletion breakpoints are shown.

We generated a CMV RNA2 construct tagged with GFP by engineering the 2b ORF region of pCMV-GTN-RNA2 (Fig. 1A), using a previously described strategy^19,21^. The engineered construct, named pCMV-GTN-RNA2-GFP, contained an intact 2a ORF and an ORF encoding the N-terminal two-thirds of 2b fused in-frame with the FMDV 2A sequence and GFP. To evaluate the infectivity and GFP expression of pCMV-GTN-RNA2-GFP, we inoculated *N. benthamiana* plants with a mixture of *Agrobacterium* cultures containing pCMV-GTN-RNA1, pCMV-GTN-RNA3, and pCMV-GTN-RNA2-GFP (this combination was designated as CMV-GFP). We found that CMV-GFP caused very mild symptoms in *N. benthamiana* plants, whereas wild-type (wt) CMV (pCMV-GTN-RNA1 + pCMV-GNT-RNA2 + pCMV-GTN-RNA3) caused severe symptoms including stunting and leaf malformation (Fig. 2A). Infiltrated plants were observed using a FOBI fluorescence imaging system at 5, 6, 7, 9, and 12 dpi, and representative data of at least three independent experiments are shown (Fig. 2B). We detected strong GFP fluorescence in the inoculated and upper uninoculated leaves of infiltrated plants until 7 dpi (Fig. 2B). However, no GFP fluorescence was observed on the newly emerging leaves after 9 dpi (Fig. 2B). RT-PCR analysis of the GFP integrity in the viral genome revealed that CMV-GFP progeny recovered from the upper young leaves that exhibited no GFP signal at 12 dpi did not carry the GFP gene (Fig. 2C,D). Moreover, sequence analysis of the amplified products revealed that almost the entire GFP sequence was deleted in the genome of CMV-GFP progenies recovered from the upper young leaves (Fig. 2E). Consistent with previous studies^19,21,37^, our results revealed that GFP tagging of CMV was unstable at the late infection stage, especially in systemically infected tissues, and thus unsuitable for visually tracking the systemic spread of the virus.

**Figure 3:**
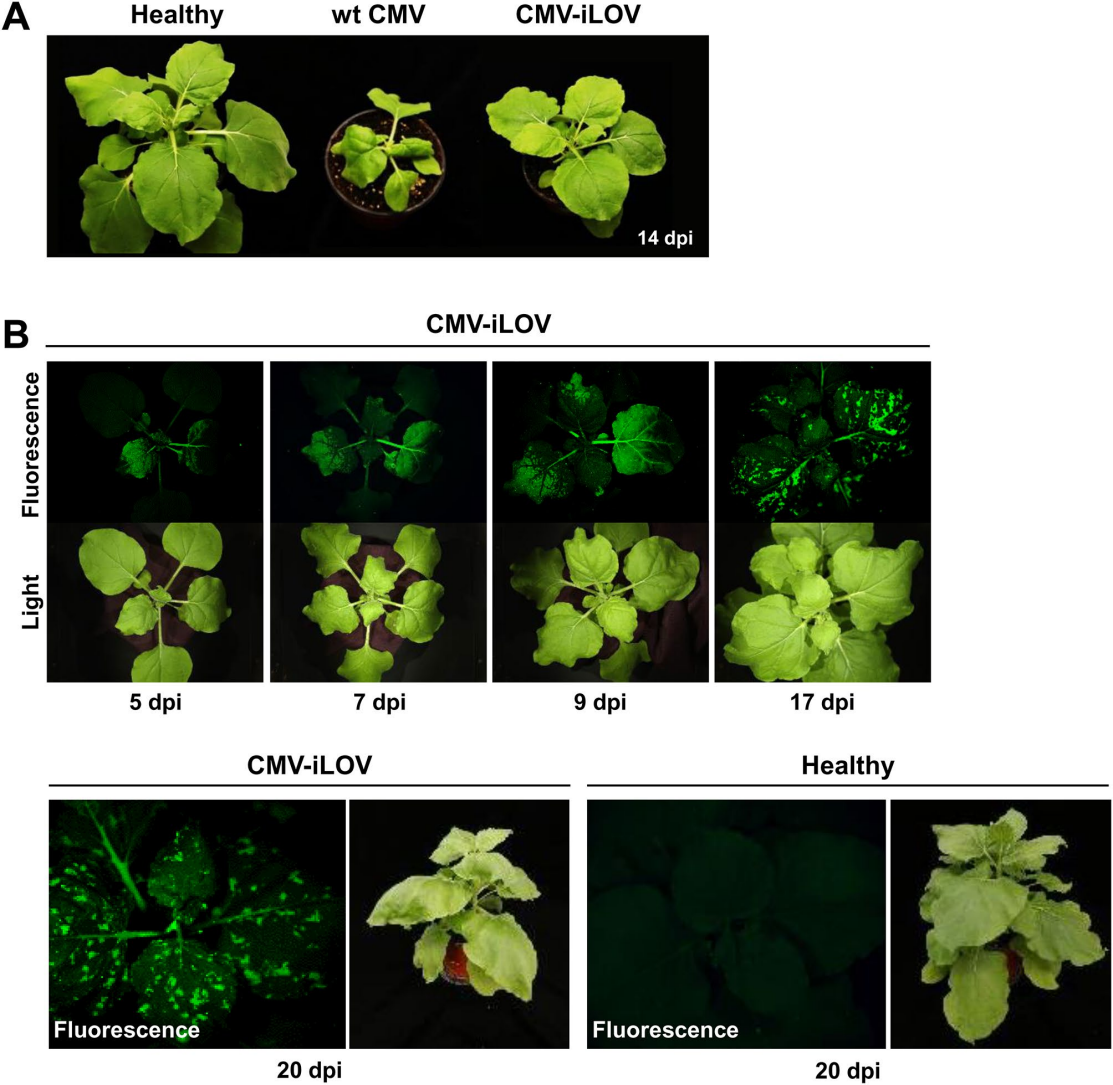
Infectivity and visual tracking of iLOV-tagged CMV (CMV-iLOV). (**A**) Virulence of infectious cDNA clones of wt CMV and CMV-iLOV in *Nicotiana benthamiana*. At 14 dpi, wt CMV induced symptoms of severe stunting and leaf malformation, whereas CMV-iLOV caused mild mosaic symptoms. (**B**) Time-course observation of CMV-iLOV infection in *N. benthamiana*. *N. benthamiana* plants inoculated with CMV-iLOV were observed using a FOBI fluorescence imaging system at 5, 7, 9, 17, and 20 dpi. Data shown are representatives of at least three independent experiments.

To reduce the genetic load caused by transgene insertion in CMV RNA2, we generated an iLOV-tagged CMV RNA2 construct by engineering the 2b ORF region (Fig. 1A) using the similar strategy as described previously^37^. The engineered construct, named pCMV-GTN-RNA2-iLOV, expressed iLOV fused to the N-terminal two-thirds of 2b. To evaluate the infectivity and iLOV expression of pCMV-GTN-RNA2-iLOV, *N. benthamiana* plants were infiltrated with a mixture of *Agrobacterium* cultures containing pCMV-GTN-RNA1, pCMV-GTN-RNA3, and pCMV-GTN-RNA2-iLOV (this combination was designated as CMV-iLOV). We found that similar to CMV-GFP, CMV-iLOV caused very mild symptoms in *N. benthamiana* plants (Fig. 3A). The plants inoculated with CMV-iLOV were observed using a FOBI fluorescence imaging system at 5, 7, 9, 17, and 20 dpi, and representative data of at least three independent experiments are shown (Fig. 3B). Consistent with the previous study^37^, we observed the systemic expression of iLOV in the inoculated plants over 20 dpi (Fig. 3B).

**Figure 4:**
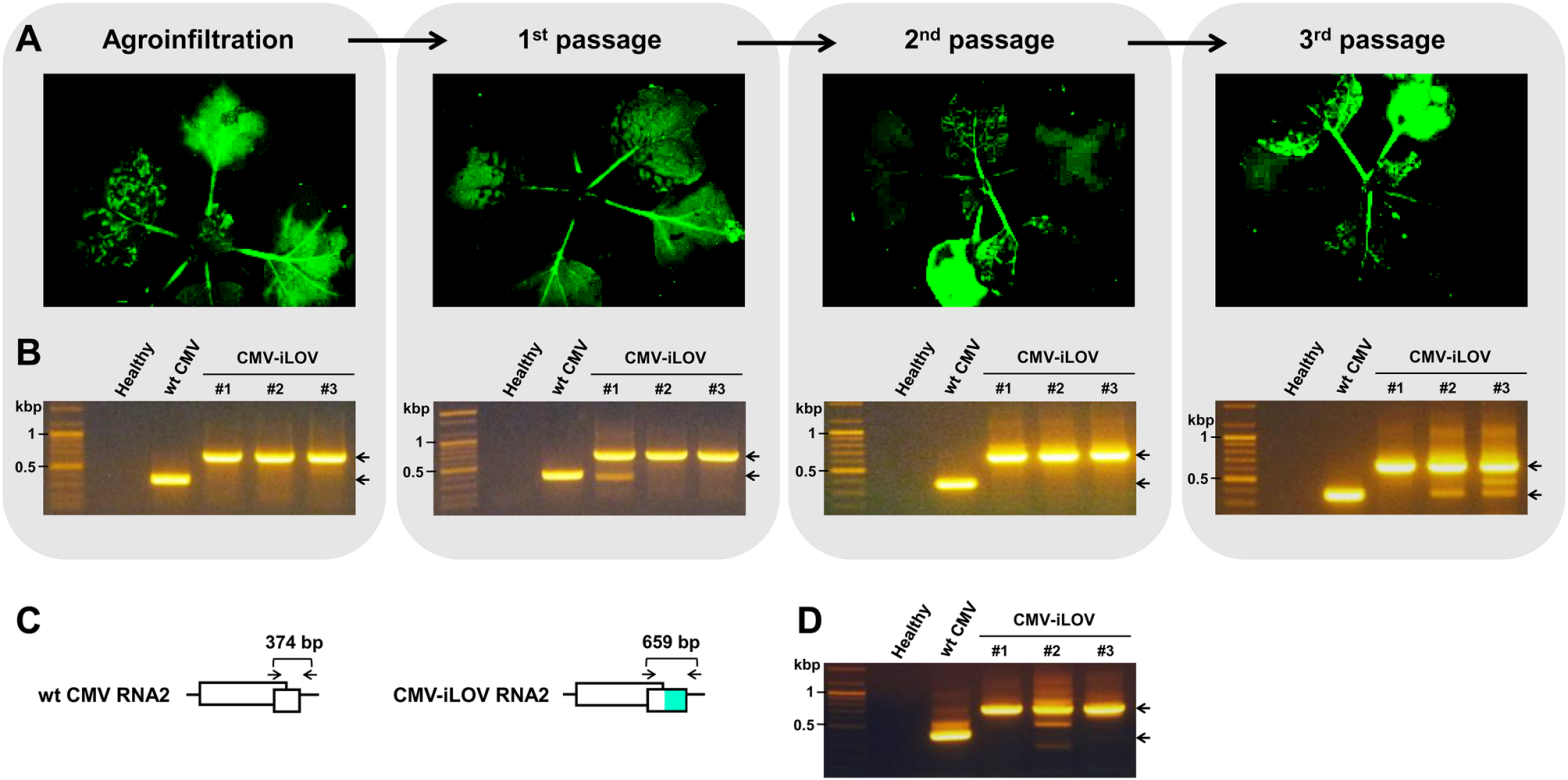
Analysis of the stability of the iLOV tag in the CMV genome. (**A**) Observation of fluorescence expression in CMV-iLOV-inoculated *N. benthamiana* plants in each passage. *N. benthamiana* plants inoculated with CMV-iLOV and its progeny viruses were observed using a FOBI fluorescence imaging system at 9 dpi. Progeny viruses were transferred thrice from plant to plant by mechanical sap-inoculation. Data shown are representatives of at least three independent experiments. (**B**) RT-PCR analysis of the integrity of CMV-iLOV. Total RNA was extracted from the upper youngest leaves of *N. benthamiana* plants inoculated with wt CMV or CMV-iLOV at 12 dpi and analyzed by RT-PCR using the primers indicated in (**C**). Numbered lanes indicate three individual plants tested in each passage. Arrows point at RT-PCR products of expected sizes, as shown in (**C**). The gels images have been cropped for clarity, and the original gels are presented in Supplementary Fig. S3. (**C**) Schematic maps of wt CMV RNA2 and CMV-iLOV RNA2. Arrows indicate the primer positions used for RT-PCR analysis, with the predicted PCR product sizes shown at the upper part. (**D**) Integrity of the iLOV tag in the CMV genome encapsidated in virions. CMV virions were purified from systemic leaves infected with CMV or CMV-iLOV at 12 dpi and subjected to RNA extraction followed by RT-PCR using the primers indicated in (**C**). Numbered lanes indicate three individual plants tested. Arrows point at RT-PCR products of expected sizes, as shown in (**C**). The gel image has been cropped for clarity, and the original gel is presented in Supplementary Fig. S3.

To test the integrity of iLOV in the viral genome, we passaged virus progeny in plant sap from plant to plant three times by mechanical inoculation. The plants inoculated with CMV-iLOV and its virus progeny were observed using a FOBI fluorescence imaging system at 9 dpi, and representative data of at least three independent experiments are shown (Fig. 4A). We readily detected iLOV fluorescence signals in all *N. benthamiana* plants inoculated with CMV-iLOV or its progeny through serial passages (Fig. 4A). Furthermore, we isolated total RNA from the upper young leaves of each individual plant and examined the iLOV integrity in the viral genome by RT-PCR using a primer pair spanning the iLOV insertion region (Fig. 4B,C). RT-PCR analysis showed the amplification of products with the predicted size for the intact CMV-iLOV (659 bp) for all *N. benthamiana* plants inoculated with CMV-iLOV or its progeny during serial passage experiments; although we also detected some products with smaller size from the plants infected with the third passage of progeny (Fig. 4B), indicating the emergence of some deletion mutant progenies. Furthermore, we examined whether iLOV-tagged RNA2 was encapsidated into virus particles. To test this, CMV virions were purified from the systemic leaves infected with CMV or CMV-iLOV at 9 dpi and subjected to viral RNA extraction followed by RT-PCR. RT-PCR analysis validated that iLOV-tagged RNA2 was efficiently packaged into virus particles (Fig. 4D). These results suggest that iLOV was highly stable in the recombinant CMV RNA2, and that CMV-iLOV can be employed for long-term visual tracking of viral infection dynamics.

### Time-course observation of CMV infection dynamics

**Figure 5:**
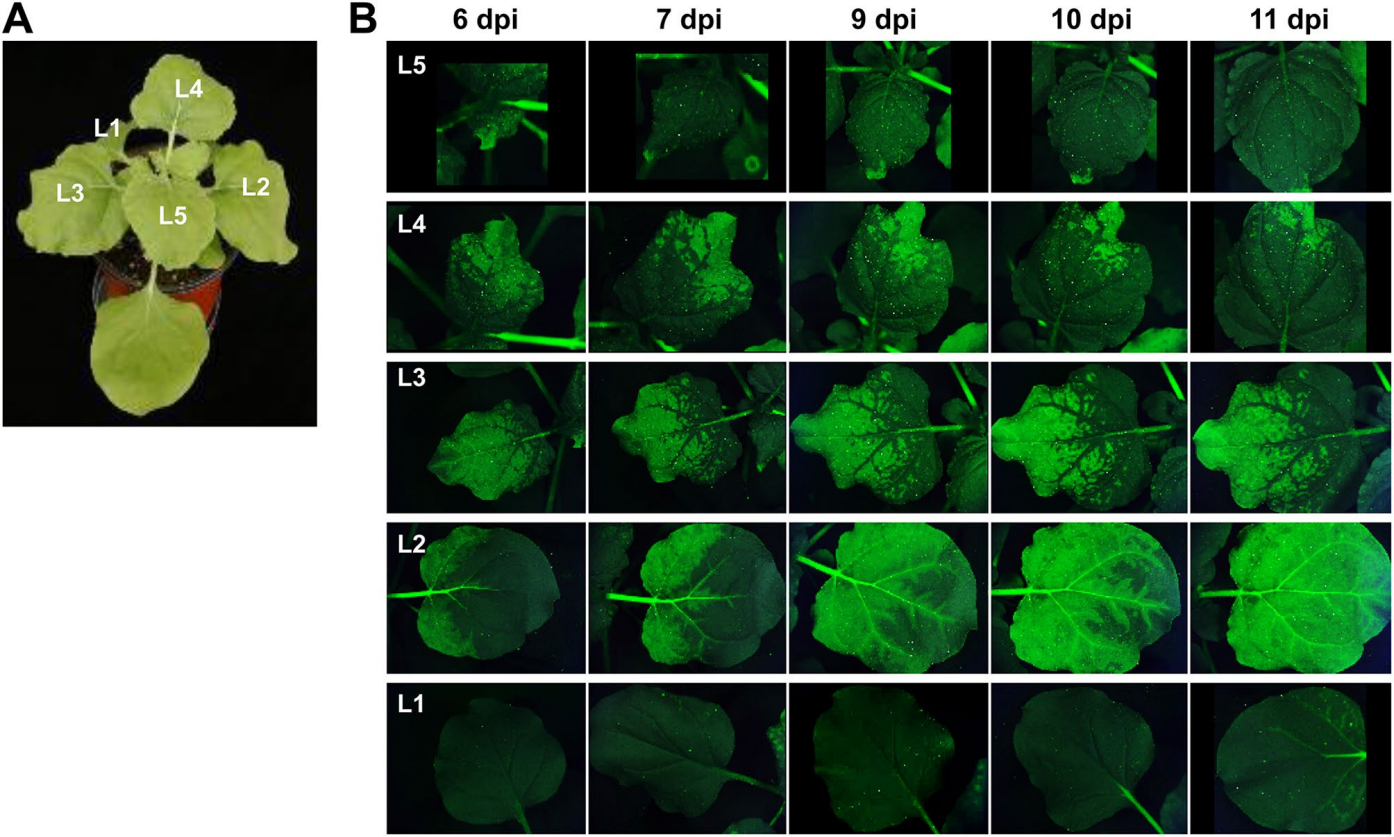
Time-course observation of CMV-iLOV infection dynamics. (**A**) A *N. benthamiana* plant infected with CMV-iLOV. Leaf positions were sequentially designated from L0 (inoculated leaves) to L5. The plant was photographed at 11 dpi. (**B**) Time-course observation of CMV-iLOV movement in differently positioned leaves. Each leaf of *N. benthamiana* plants inoculated with CMV-iLOV was observed using a FOBI fluorescence imaging system at 6, 7, 9, 10, and 11 dpi. Data shown are representatives of at least three independent experiments.

To examine the infection dynamics of CMV at the whole plant level, we inoculated three *N. benthamiana* plants with CMV-iLOV and observed them using a FOBI fluorescence imaging system at different times post-inoculation (6, 7, 9, 10, and 11 dpi), and representative data of at least three independent experiments are shown (Fig. 5). The leaf positions in inoculated plants were designated sequentially from L0 (inoculated leaves) to L5. At 6 dpi, CMV infection was observed in the basal part of L2, in approximately the entire part of L3, in the upper half part of L4, and in the apex part of L5 of CMV-iLOV-inoculated plants (Fig. 5). We also noticed that the virus-infected area in L2 was subsequently expanded along major veins from the basal part to the upper area over time (Fig. 5). At 11 dpi, the entire part of L2 was infected with CMV-iLOV. In contrast, we did not observe any further expansion of the virus-infected area in L3, L4, and L5 (Fig. 5). In particular, the virus remained only in the apex part of L5 despite its rapid growth. In L3, the virus was unable to invade major veins on the lower half of the leaf (Fig. 5). No fluorescence signal was observed in L1 until 9 dpi. However, we detected iLOV signals in the petiole of L1 at 10 dpi, and observed viral spreading along major veins on the basal part of L1 at 11 dpi (Fig. 5).

### Time-course observation of BBWV2 infection dynamics

**Figure 6:**
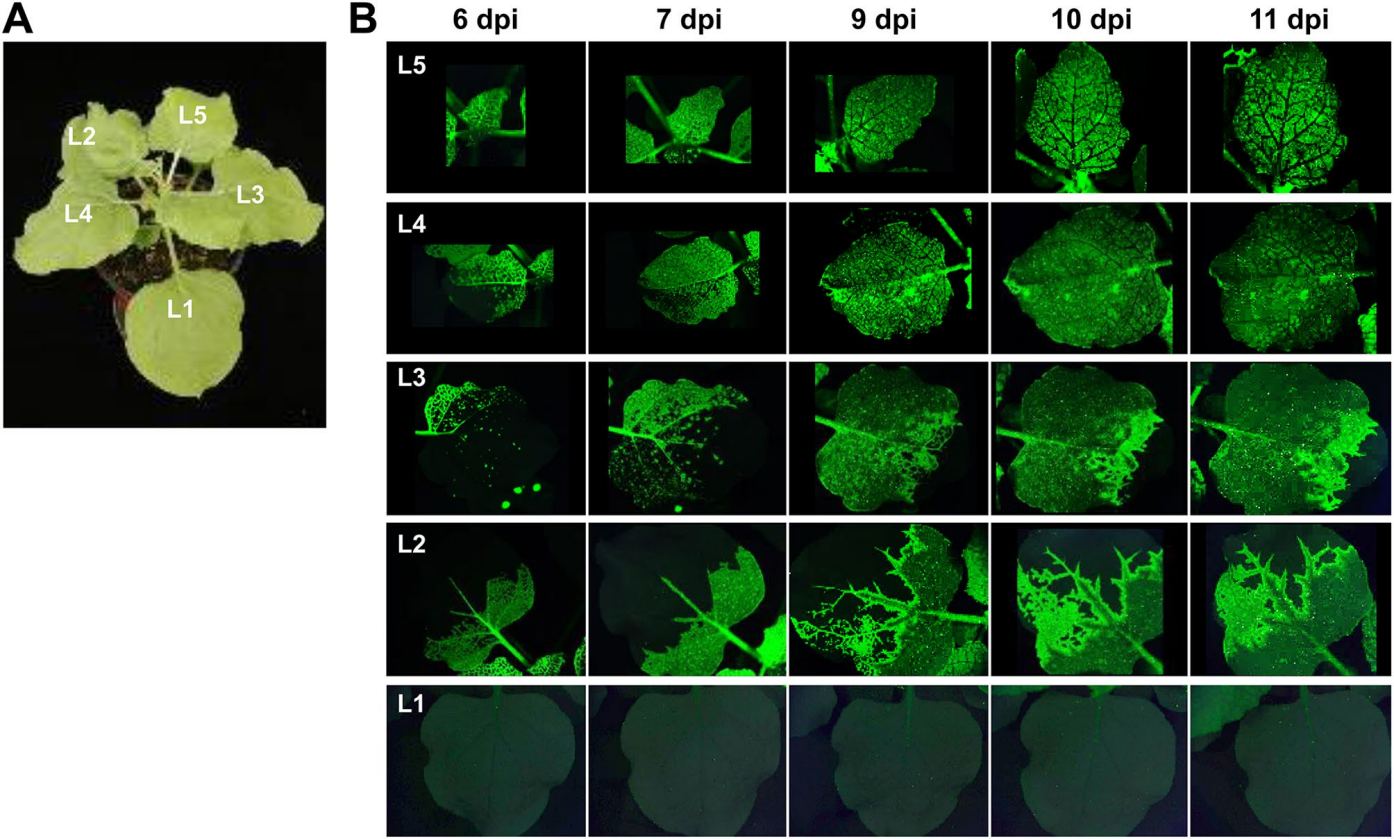
Time-course observation of BBWV2-GFP infection dynamics. (**A**) A *N. benthamiana* plant infected with BBWV2-GFP. Leaf positions were sequentially designated from L0 (inoculated leaves) to L5. The plant was photographed at 11 dpi. (**B**) Time-course observation of BBWV2-GFP movement in differently positioned leaves. Each leaf of *N. benthamiana* plants inoculated with BBWV2-GFP was observed using a FOBI fluorescence imaging system at 6, 7, 9, 10, and 11 dpi. Data shown are representatives of at least three independent experiments.

We first investigated the infection dynamics of BBWV2 in *N. benthamiana* plants before examining whether co-infection with BBWV2 affects CMV infection dynamics. We inoculated three *N. benthamiana* plants with BBWV2-GFP, a mixture of Agrobacterium cultures containing pBBWV2-RP1-R1 + pBBWV2-R2-OE-GFP^30^ and observed them for GFP signals at different times post-inoculation (6, 7, 9, 10, and 11 dpi), and representative data of at least three independent experiments are shown (Fig. 6). At 6 dpi, BBWV2 infection was observed in the basal parts of L2 and L3, in the lower half part of L4, and in the entire part of L5 (Fig. 6). Thus, we hypothesized that BBWV2 spread faster to the upper young leaves during viral long- distance movement. In L2, L3, and L4, BBWV2 infection expanded from the basal parts to the upper areas along veins over time (Fig. 6). At 11 dpi, in contrast to CMV-iLOV, BBWV2-GFP infected the entire parts of L4 and L5, and the movement of the virus to the upper area was still in progress in L2 and L3. However, we did not observe any BBWV2 infection in L1 at 11 dpi and beyond (Fig. 6).

### Effects of co-infection with BBWV2 on CMV infection dynamics

**Figure 7:**
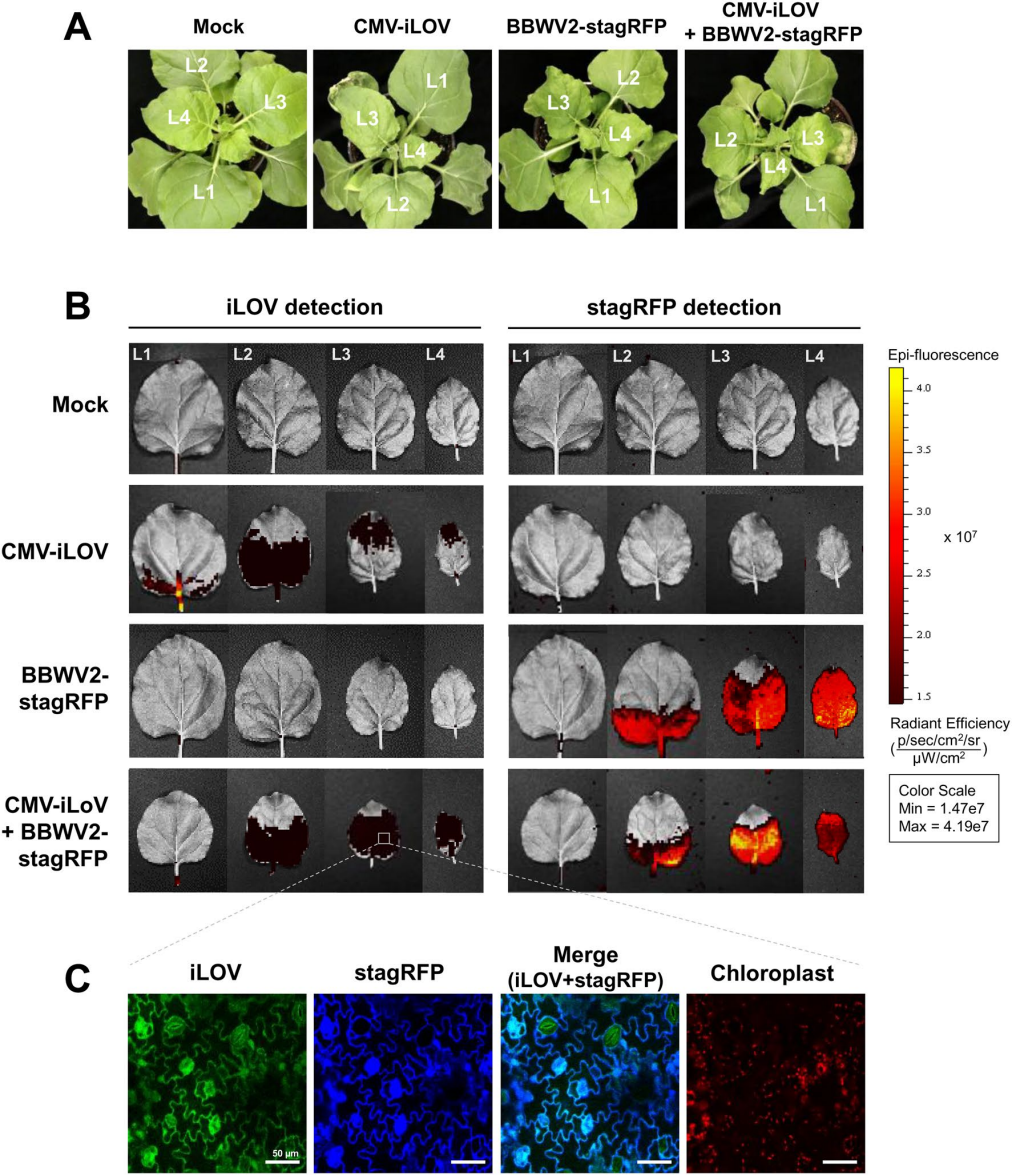
Effects of BBWV2 co-infection on CMV infection in *N. benthamiana*. (**A**) Virulence of CMV-iLOV and BBWV2-stagRFP in single or mixed infection in *N. benthamiana*. At 10 dpi, plants infected with either CMV-iLOV or BBWV2-stagRFP exhibited typical mild symptoms, whereas those co-infected with both viruses developed more severe symptoms of mosaic and leaf size reduction. Leaf positions were sequentially designated from L0 (inoculated leaves) to L4. (**B**) Visual detection of CMV-iLOV and BBWV2-stagRFP in single or mixed infection in differently positioned leaves. Each leaf of *N. benthamiana* plants infected with either CMV-iLOV or BBWV2-stagRFP or both was observed using an IVIS Lumina III fluorescence imaging system at 10 dpi. Data shown are representatives of at least three independent experiments. (**C**) Confocal microscopic observation of leaf tissues (boxed area in **B**) co-infected with CMV-iLOV or BBWV2-stagRFP. Bar = 50 μm.

We next examined whether CMV infection dynamics was influenced by co-infection with BBWV2. We inoculated *N. benthamiana* plants with inoculation buffer (mock), CMV-iLOV, BBWV2-stagRFP, or a mixture of CMV-iLOV and BBWV2-stagRFP. At 10 dpi, the inoculated plants were observed for symptom development and fluorescence expression using an IVIS Lumina III fluorescence imaging system, and representative data of at least three independent experiments are shown (Fig. 7). We found that plants inoculated with either CMV-iLOV or BBWV2-stagRFP exhibited typical symptoms, whereas co-inoculation with the two viruses caused a distinguishable reduction in leaf size (Fig. 7A,B). We observed iLOV and stagRFP signals on L1-L4 leaf positions. Compared with BBWV2 single infection, BBWV2 infection dynamics seemed to not be influenced by co-infection with CMV (Fig. 7B). However, CMV infection dynamics was significantly affected by co-infection with BBWV2. In particular, approximately the entire parts of L3 and L4 were infected with CMV in the plants co-infected with CMV and BBWV2, whereas CMV infection was only observed in the upper apex parts of L3 and L4 of the plants infected with CMV alone (Fig. 7B).

We next aimed to examine whether spatial interference occurs between CMV and BBWV2 at the cellular level when the two viruses simultaneously infect the same leaf area. To this end, we observed the L3 leaf tissues showing both iLOV and stagRFP signals (boxed area in Fig. 7B) using confocal microscopy. The observed cells simultaneously expressed both iLOV and stagRFP signals throughout the cytoplasm (Fig. 7C), indicating that CMV and BBWV2 co-infected the same cells in the systemic leaves.

**Figure 8:**
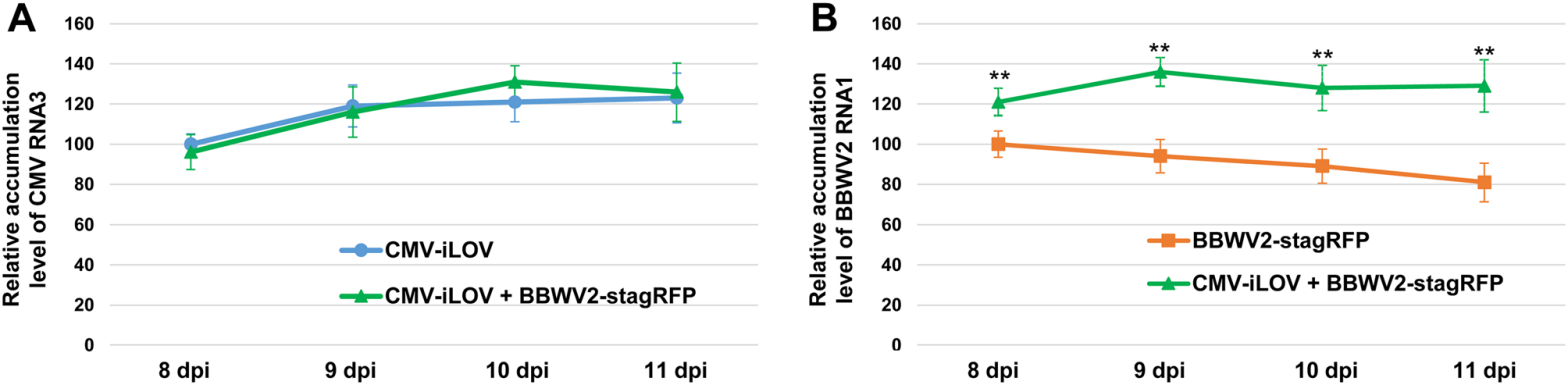
Accumulation levels of CMV-iLOV and BBWV2-stagRFP in single or mixed infection. Total RNA was isolated from the basal parts of L2 (expressing either or both iLOV and stagRFP) of three individual plants infected with either or both viruses at 8, 9, 10, and 11 dpi, and subjected to RT-qPCR to analyze the relative accumulation levels of CMV RNA3 (**A**) and BBWV2 RNA1 (**B**) in single or mixed infection. The mean ± SD of three replications are shown and each column represents one group with nine plants. Significant differences were analyzed using a paired Student's *t*-test (**P < 0.01).

We also examined whether co-infection with the two viruses affects the accumulation level of each virus in plants. Total RNA was isolated from the basal parts of L2 (expressing either one or both iLOV and stagRFP) of plants infected with CMV-iLOV and/or BBWV2-stagRFP at 8, 9, 10, and 11 dpi and analyzed by RT-qPCR. The accumulation level of CMV-iLOV was not significantly affected by co-infection with BBWV2-stagRFP (Fig. 8A). In contrast, the accumulation level of BBWV2-stagzRFP in the leaf tissues co-infected with CMV-iLOV and BBWV2-stagRFP increased significantly compared with that in the leaf tissues infected with only BBWV2-stagRFP (Fig. 8B).

## Discussion

For accurate tracking of a virus tagged with a reporter gene, the first issue that should be addressed is the integrity of the inserted reporter gene in the viral genome. As viruses have evolved to optimize their genome fitness, artificially inserted foreign sequences are easily eliminated from viral genomes during robust replication^36,46,47^. Therefore, the size of a reporter gene acts as a constraint on the integrity of a tagged recombinant virus^48^. In this regard, tagging CMV with iLOV (375 bp), which is a considerably smaller alternative to GFP (~ 720 bp), could reduce the genetic load and be less detrimental to viral fitness, including replication, movement, encapsidation, and transmission. More specifically, iLOV-tagged CMV RNA2 (3330 nt) is 285 nt longer than CMV-GTN wt RNA2 but 28 nt shorter than CMV-GTN RNA1. As seen, the encapsidation of iLOV-tagged CMV RNA2 was not hindered by the physical limitations of viral particle assembly imposed by genome fragment size (Fig. 4D). Further, unlike CMV tagged with GFP or DsRed2^19,37^, CMV-iLOV retained the iLOV gene after 28 dpi in systemically infected leaves^37^. Our results also revealed that the integrity of CMV-iLOV was maintained during serial passage experiments in which sap from infected tissues was used as an inoculum (Fig. 4A,B). The outperformance of iLOV as a reporter of viral infection was also shown in visualizing TMV infection in *N. tabacum*^32^. Specifically, iLOV-tagged TMV caused pervasive infection in both inoculated and systemic leaves, whereas GFP-tagged TMV caused restricted infection in the inoculated leaves and significantly decreased systemic infection^32^. The enhanced functionality of iLOV might be attributed to the reduced genetic load on viral fitness.

Previous studies showed that GFP-tagging at the C-terminus of the truncated 2b had little effect on the disease symptoms in *N. benthamiana* plants when compared with to wt viruses^19,21^. In addition, truncation or GFP fusion at the C-terminus of the 2b protein was shown to not have any significant effects on the RNA silencing suppressor activity^19,49^. However, in this study, *N. benthamiana* plants infected with CMV-GFP or CMV-iLOV exhibited very mild symptoms compared with those infected with the wt virus (Figs. 2A, 4A). Although one possibility for these contradictory observations may be that we used a CMV strain (GTN) different from those (Fny and Y strains) used previously^19,21^, our results suggested that the C-terminal one-third region of 2b might contain motifs associated with symptom severity in CMV infection.

When we examined CMV infection dynamics in *N. benthamiana* using CMV-iLOV, we observed that the virus initially infected the apex part of the upper young leaves, but did not spread after a certain time point (Fig. 5). However, in the lower expanded leaves, the viral infection began at the basal part and subsequently spread upwards along major veins over time (Fig. 5). The impaired movement of CMV-iLOV in the upper young leaves was likely associated with a reduction in symptom severity in *N. benthamiana* plants infected with the virus. CMV 2b functions as an RNA silencing suppressor, but was also shown to affect the local and systemic movement of the virus^10,11,50^. However, the influence of 2b on CMV movement is likely viral strain-specific; a mutant of the CMV Fny strain that did not express the entire 2b protein (Fny-CMVΔ2b) was able to systemically infect *N. tabacum* and *N. benthamiana* but did not induce symptoms^51,52^, whereas the 2b deletion mutation in the CMV Q strain resulted in decreased symptom severity in *N. tabacum* and *N. glutinosa*^53^. Meanwhile, *Cucurbita pepo* plants infected with Fny-CMVΔ2b showed mild symptoms early in infection, but symptom recovery on upper young leaves^54^. Symptom recovery, which is characterized by the emergence of asymptomatic leaves after an initial symptomatic infection in lower leaves, is a natural phenomenon in some plant–virus interactions^55,56^. In various virus–plant interactions, symptom recovery was demonstrated to be associated with antiviral RNA silencing^55^. The induction of antiviral RNA silencing can result in reduced virus titers and mild or no symptoms in the recovered leaves. CMV induces a specific symptom recovery phenotype, known as cyclic mosaic symptoms in *N. tabacum* plants^57,58^. In CMV, defects in the RNA silencing activity of the 2b protein were shown to result in symptom recovery^58^. Thus, combining our results with those of previous studies^57–59^, we suggest that symptom recovery in CMV-infected plants was likely caused by impaired viral movement in upper young leaves as well as reduced viral titers, and associated with the suppression of the activities of 2b.

Mixed infections of related or unrelated viruses cause various intrahost virus–virus interactions^60–62^. These interactions between viruses in mixed infections are generally categorized as synergistic or antagonistic. Moreover, synergistic and antagonistic interactions are likely to occur simultaneously in mixed infections of some viruses, resulting in unpredictable biological and epidemiological outcomes^19,63^. Synergistic interactions are usually accompanied by an enhancement of disease symptoms and an increase in the titer of one or both viruses, whereas antagonistic interactions prevent the subsequent infection of closely related viruses and result in the spatial separation of co-infecting viruses^62,64–66^. Thus far, the molecular and cellular mechanisms underlying synergistic or antagonistic interactions between viruses in mixed infections have been partially understood or remain hypothetical. Visual tracking of the infection dynamics of viruses using fluorescent tags is required to further detailed studies on intrahost virus–virus interactions.

In mixed infection of plants with CMV-iLOV and BBWV2-stagRFP, we observed that CMV-iLOV spread throughout all leaf tissues in the upper young leaves, indicating that BBWV2 facilitated the cell-to-cell movement of CMV (Fig. 7B). Unlike CMV, the cell-to-cell movement of BBWV2 seemed to not be affected by co-infection with CMV (Fig. 7B). CMV and BBWV2 utilize different cell-to-cell movement mechanisms. CMV is able to move through plasmodesmata (PD) as a viral ribonucleoprotein (RNP) complex, while the ability of the CMV MP to generate tubule structures in the PD is not obligatory for cell-to-cell movement^2,67^. In contrast, BBWV2 moves as a spherical virion through MP-induced tubule structures in the PD^68^. Viral MP-induced tubules cause large increases in the size exclusion limit of the PD^69^. Thus, BBWV2-induced tubule structures might facilitate the cell-to-cell movement of CMV virions or RNA complexes.

The synergistic interactions between CMV and BBWV2 were also observed in increases in symptom severity and BBWV2 accumulation in co-infected tissues (Figs. 7, 8). An increase in accumulation of abutilon mosaic virus (AbMV; genus *Begomovirus*) in AbMV-CMV synergism was shown to be due to the 2b protein^70^. The 2b protein has also been shown to function in some synergistic interactions between CMV and potyviruses, including turnip mosaic virus and zucchini yellow mosaic virus^19,54^. Confocal microscopic observation revealed that CMV and BBWV2 actively replicated in the same cells, indicating a lack of spatial interference between the two viruses, and the 2b protein influenced the accumulation of BBWV2 (Fig. 7C). The RNA silencing suppressor activity of the C-terminally truncated 2b protein was found to be similar to that of the intact 2b protein^19,49^. Thus, the 2b protein might play a crucial role in the synergistic interactions between CMV and BBWV2, including symptom induction and viral RNA accumulation in co-infected tissues.

Using fluorescently tagged viruses, we showed that CMV and BBWV2 synergistically interact when co-infecting a plant host: (i) the enhancement of the CMV cell-to-cell movement in upper young leaves (thereby, the two viruses infect the same leaf areas); (ii) an increase in BBWV2 accumulation; (iii) an increase in symptom severity; and (iv) active replication in the same cells without spatial interference. This synergism in CMV and BBWV2 infection dynamics might be linked to their transmission dynamics because both viruses can be transmitted by the same aphid vectors^39,40^. Previously, field surveys have reported that CMV and BBWV2 are highly prevalent in pepper fields in South Korea and that the incidence of mixed infection by the two viruses is higher than 50%^3,38^. While further in-depth studies are required to understand the basis of synergistic interactions between CMV and BBWV2 in vector-mediated transmission dynamics, fluorescently tagged recombinant viruses, such as CMV-iLOV and BBWV2-stagRFP, can be used as tools for the examination of viral transmissibility and virus-vector interactions under single and mixed infection conditions.

## Supplementary Information

Supplementary Figure S1.

Supplementary Figure S2.

Supplementary Figure S3.

## Data Availability

The datasets generated during and/or analyzed during the current study are available from the corresponding author on reasonable request.
