# Supplementary material for: Visual tracking of viral infection dynamics reveals the synergistic interactions between cucumber mosaic virus and broad bean wilt virus 2
Source: Sci Rep. 2023 May 4;13:7261. doi: 10.1038/s41598-023-34553-6 (PMC10160061; doi:10.1038/s41598-023-34553-6)
Supplement: Supplementary file 1 — Supplementary Figure S1. [file 41598_2023_34553_MOESM1_ESM.pdf]

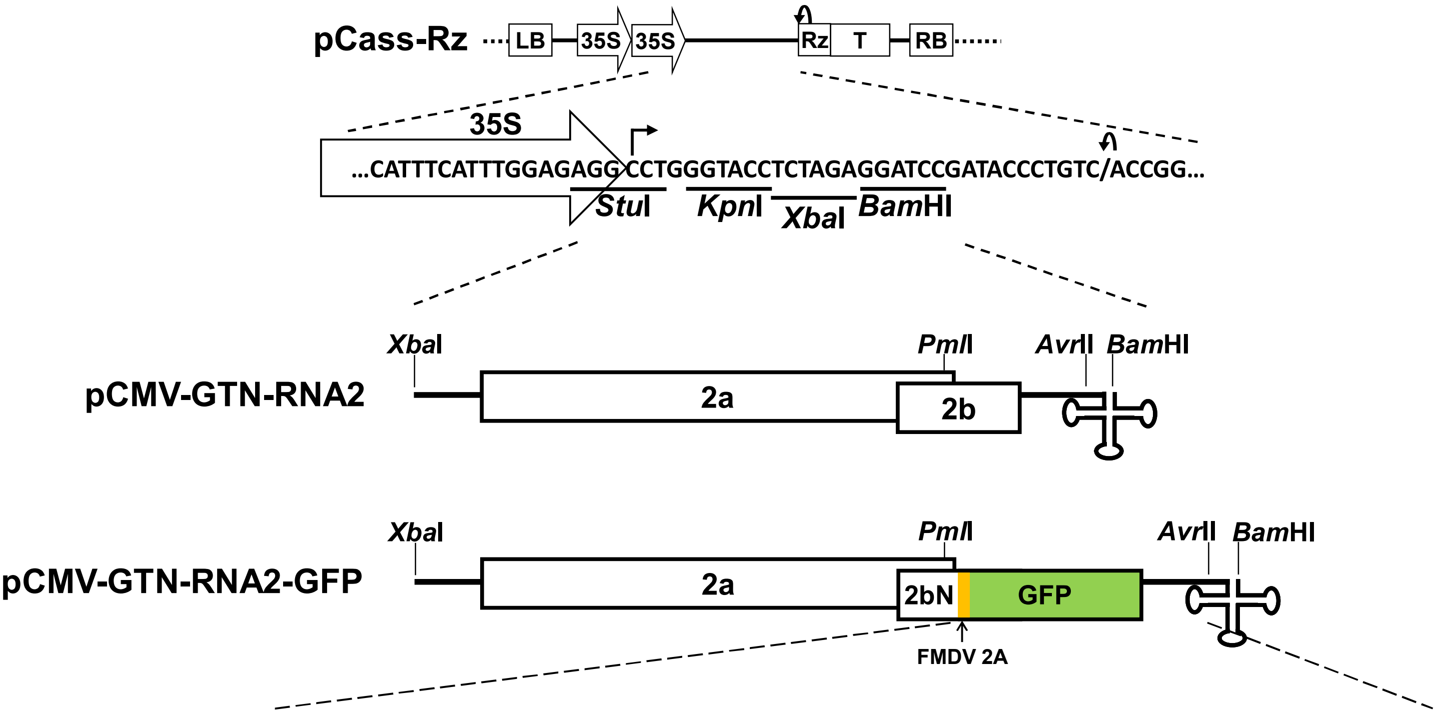

CACGTGAACGTGGCGGAATTGTCCGAGTCTGAGCTGCTAAACTTCGATCTCTTGAAGCTTGCA
GGAGATGTTGAGTCAAATCCAGGTCCAACGCGTATGGTGAGCAAGGGCGAGGAGCTGTTCACC
GGGGTGGTGCCCATCCTGGTCGAGCTGGACGGCGACGTAAACGGCCACAAGTTCAGCGTGTC
CGGCGAGGGCGAGGGCGATGCCACCTACGGCAAGCTGACCCTGAAGTTCATCTGCACCACCG
GCAAGCTGCCCGTGCCCTGGCCCACCCTCGTTACCACCTTCACCTACGGCGTGCAGTGCTTCA
GCCGCTACCCCGACCACATGAAGCAGCACGACTTCTTCAAGTCCGCCATGCCCGAAGGCTACG
TCCAGGAGCGCACCATCTTCTTCAAGGACGACGGCAACTACAAGACCCGCGCCGAGGTGAAG
TTCGAGGGCGACACCCTGGTGAACCGCATCGAGCTGAAGGGCATCGACTTCAAGGAGGACGG
CAACATCCTGGGGCACAAGCTGGAGTACAACTACAACAGCCACAACGTCTATATCATGGCCGAC
AAGCAGAAGAACGGCATCAAGGTGAACTTCAAGATCCGCCACAACATCGAGGACGGCAGCGT
GCAGCTCGCCGACCACTACCAGCAGAACACCCCCATCGGCGACGGCCCCGTGCTGCTGCCC
GACAACCACTACCTGAGCACCCAGTCCGCCCTGAGCAAAGACCCCAACGAGAAGCGCGATCA
CATGGTCCTGCTGGAGTTCGTGACCGCCGCCGGGATCACTCACGGCATGGACGAGCTGTACA
AGGGGATCCTCTGAACGCGTACTAGTGACTACAAGGACGACGATGACAAGGCAGATTATAAAGA
TGATGACGATAAACATCATCACCATCACCACTGAACCCCTCCTTCCTTTCTCCCTCCAGTTTTCT
GAGGCGGGAGCTGAGTTGGCAGTATTGCTACAAACTGTCTGAAGTCACTAAACGTTTTACGGTG
AACGGGTTGTCCATCCAGCTAACGGCTAAAATGGTCAGTCGCGGAGAAATCCACGCCGGAGAT
TTACAAGTCTCTGAGGCACCTTTGAAACCATCTCCTAGG

Supplementary Fig. S1. Construction of pCMV-GTN-RNA2-GFP. A 1106-bp DNA fragment consisting of the C-terminus of 2a (from the *Pml*I site), the 2A self-cleaving sequence of foot and mouse disease virus (FMDV), an *MluI* site, the GFP coding sequence, and the viral 3' untranslated region (UTR) (to the *Avr*II site) was synthesized and inserted into pCMV-GTN-RNA2, which was opened with *Pml*I and *Avr*II. The resulting construct was named pCMV-GTN-RNA2-GFP. *Pml*I and *Avr*II sites are underlined in the sequence.
