# Supplementary material for: Visual tracking of viral infection dynamics reveals the synergistic interactions between cucumber mosaic virus and broad bean wilt virus 2
Source: Sci Rep. 2023 May 4;13:7261. doi: 10.1038/s41598-023-34553-6 (PMC10160061; doi:10.1038/s41598-023-34553-6)
Supplement: Supplementary file 2 — Supplementary Figure S2. [file 41598_2023_34553_MOESM2_ESM.pdf]

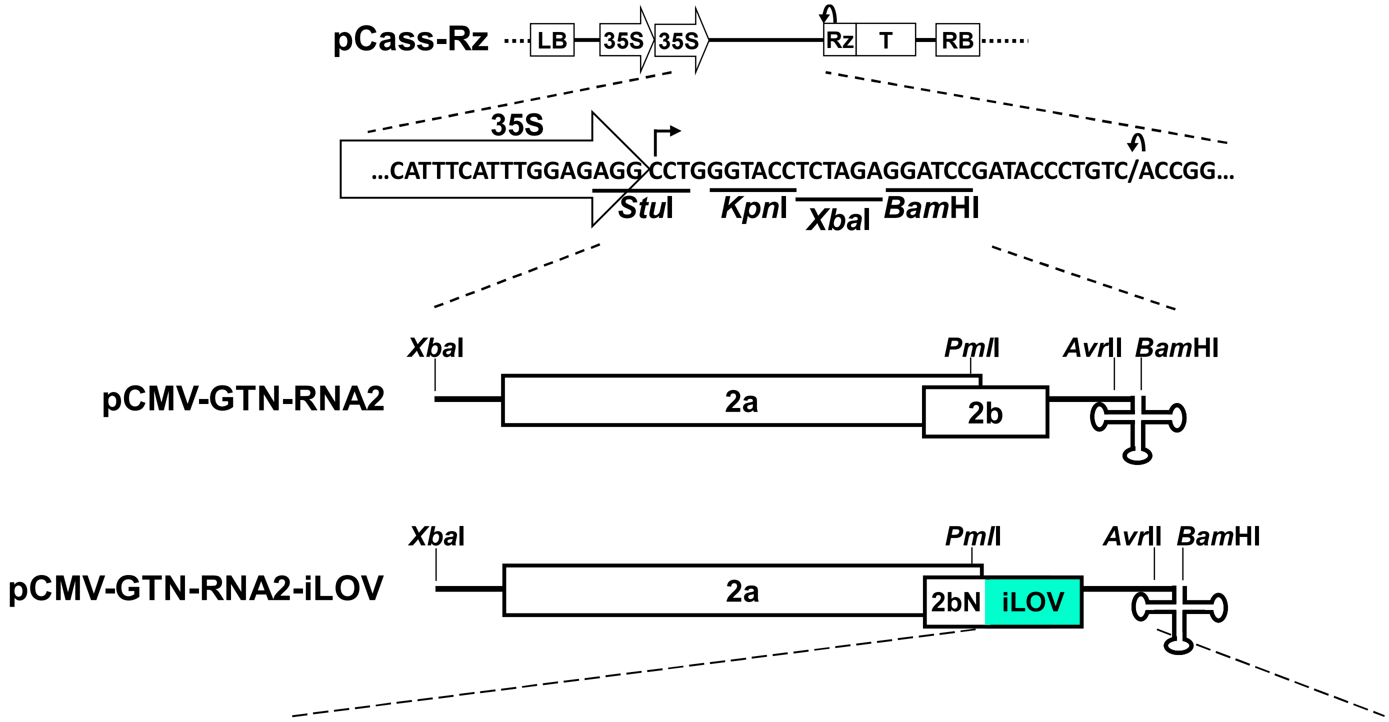

CACGTGAACGTGGCGGAATTGTCCGAGTCTGAGATGATCGAAAAGAATTTCGTCATCAC
CGATCCACGACTTCCAGATAACCCCATTATATTCGCATCTGATGGTTTTCTCGAACTTACT
GAATACAGTAGGGAAGAAATACTTGGTAGAAATGCTCGTTTTCTCCAAGGACCAGAGACA
GATCAGGCAACTGTGCAAAAGATAAGGGACGCAATTAGAGACCAGCGGGAGACTACCG
TACAACTTATTAATTACACAAAGTCTGGCAAAAAGTTCTGGAATCTCCTGCACCTGCAGC
CTGTCAGAGATCAGAAAGGCGAGTTGCAGTATTTTATTGGAGTCCAGTTGGACGGGAGT
GACCATGTCTCCGGTGGTGGAGGCTCAGATTATAAGGATGATGATGATAAGTGAACCCCT
CCTTCCTTTCTCCCTCCAGTTTTCTGAGGCGGGAGCTGAGTTGGCAGTATTGCTACAAA
CTGTCTGAAGTCACTAAACGTTTTACGGTGAACGGGTTGTCCATCCAGCTAACGGCTAAA
ATGGTCAGTCGCGGAGAAATCCACGCCGGAGATTTACAAGTCTCTGAGGCACCTTTGAA
ACCATCTCCTAGG

Supplementary Fig. S2. Construction of pCMV-GTN-RNA2-iLOV. A 608-bp DNA fragment consisting of the C-terminus of 2a (from the *Pml*I site), the iLOV coding sequence, and the viral 3' UTR (to the *Avr*II site) was synthesized and inserted into pCMV-GTN-RNA2, which was opened with *Pml*I and *Avr*II. The resulting construct was named pCMV-GTN-RNA2-iLOV. *Pml*I and *Avr*II sites are underlined in the sequence.
