# Supplementary figures and images for: Visual tracking of viral infection dynamics reveals the synergistic interactions between cucumber mosaic virus and broad bean wilt virus 2
Source: Sci Rep. 2023 May 4;13:7261. doi: 10.1038/s41598-023-34553-6 (PMC10160061; doi:10.1038/s41598-023-34553-6)

Fig. 2D

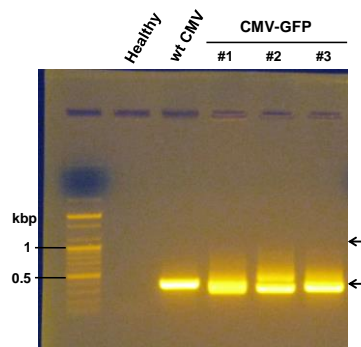

Fig. 4B

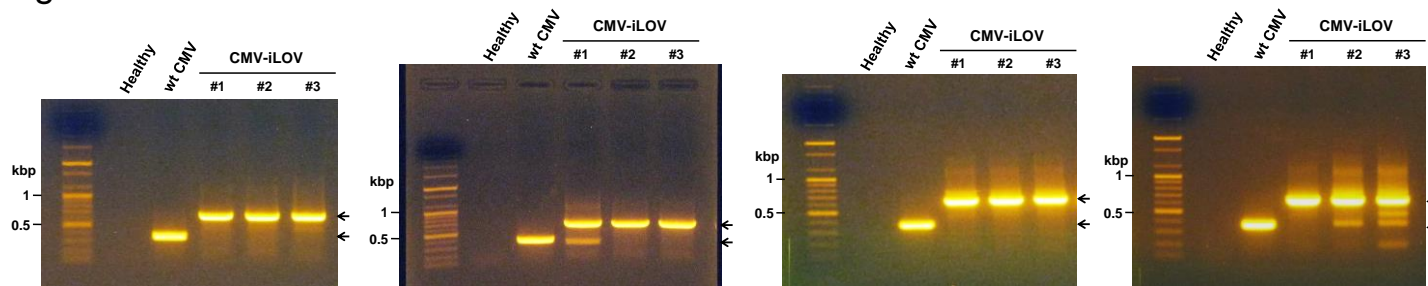

Fig. 4D

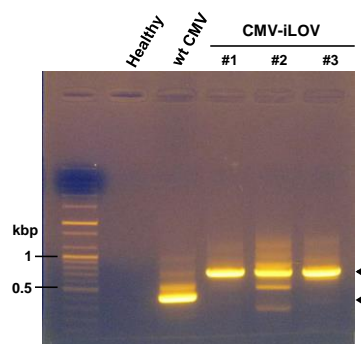

Supplement: Supplementary file 3 — Supplementary Figure S3. [file 41598_2023_34553_MOESM3_ESM.pdf]
